# Three-Dimensional Double-Layer Multi-Stage Thermal Management Fabric for Solar Desalination

**DOI:** 10.3390/ma17174419

**Published:** 2024-09-07

**Authors:** Xiao Feng, Can Ge, Heng Du, Xing Yang, Jian Fang

**Affiliations:** 1 College of Textile and Clothing Engineering, Soochow University, Suzhou 215123, China; 19930548047@163.com (X.F.); gecan1999gc@163.com (C.G.); 15982845916@163.com (H.D.); ys1132023@163.com (X.Y.); 2 National Engineering Laboratory for Modern Silk, Soochow University, Suzhou 215123, China

**Keywords:** photothermal conversion, desalination, solar interfacial evaporation, thermal management, fabric

## Abstract

Water scarcity is a serious threat to the survival and development of mankind. Interfacial solar steam generation (ISSG) can alleviate the global freshwater shortage by converting sustainable solar power into thermal energy for desalination. ISSG possesses many advantages such as high photothermal efficiency, robust durability, and environmental friendliness. However, conventional evaporators suffered from huge heat losses in the evaporation process due to the lack of efficient thermal management. Herein, hydrophilic Tencel yarn is applied to fabricate a three-dimensional double-layer fabric evaporator (DLE) with efficient multi-stage thermal management. DLE enables multiple solar absorptions, promotes cold evaporation, and optimizes thermal management. The airflow was utilized after structure engineering for enhanced energy evaporation efficiency. The evaporation rate can reach 2.86 kg·m^−2^·h^−1^ under 1 sun (1 kW·m^−2^), and 6.26 kg·m^−2^·h^−1^ at a wind speed of 3 m·s^−1^. After a long duration of outdoor operation, the average daily evaporation rate remains stable at over 8.9 kg·m^−2^, and the removal rate of metal ions in seawater reaches 99%. Overall, DLE with efficient and durable three-dimensional multi-stage thermal management exhibits excellent practicality for solar desalination.

## 1. Introduction

Freshwater scarcity has been a serious impediment to the path of sustainable development over the past few decades as a result of increasing population and growing demand [1,2,3]. Access to clean water is essential for the continued survival and development of humankind. However, seawater accounts for 97% of global water resources, with only 3% of water resources in the form of freshwater [4,5]. The traditional desalination methods including membrane distillation, reverse osmosis, and electrodialysis require extra energy consumption and complex equipment [6,7,8]. The environmentally friendly, efficient, and convenient interfacial solar steam generation (ISSG) can convert clean solar energy into thermal energy [9], purifying seawater at the gas–liquid interface. Afterward, clean freshwater can be directly obtained through condensation and purification [10]. The ISSG enables green and sustainable desalination through uncomplicated and low-cost processes [11].

During the ISSG process, heat energy is inevitably lost due to thermal radiation, reflection, convection, and conduction [12]. Fibrous materials show great competitiveness in the ISSG field due to ideal processability, good structural plasticity, diverse varieties, and convenient manufacturing [13]. Compared to rigid materials, flexible fibrous materials facilitate the processing of evaporators and the loading of various photothermal materials. The low thermal conductivity of the fibrous material inhibits heat loss, and the high porosity of fabrics is conducive to steam escape [14]. For example, Fan [15] used hydrophilic materials as warp yarns and flexible polyaniline photothermal fibers as weft yarns to accurately construct an effective water supply pathway using photothermal fabrics through shuttle weaving technology. They achieved excellent water heat distribution at the solar evaporation interface, greatly improving the photothermal conversion efficiency and evaporation rate. Wang [16] designed an interfacial solar evaporator with a 3D origami structure using nanocomposites of graphene oxide and carbon nanotubes as a photothermal component. By varying the degree of origami folding, the surface area density can be controlled to increase solar absorption, and also to suppress radiative and convective heat loss from evaporating surfaces. However, the existing ISSGs have the following problems: (1) low utilization of solar energy; (2) the thermal management methods are limited to heat loss reduction and low heat utilization; (3) lack of utilization of wind energy in the environment, which severely limits their practical application [17,18,19].

In this study, a three-dimensional double-layer photothermal fabric evaporator with a hollow structure (DLE) was designed by a traditional textile processing method. A Tencel fiber with great hydrophilicity and flexibility was selected as the substrate. Graphene oxide plays the role of the photothermal conversion material with a broadband spectrum of strong solar absorption, high chemical stability, and hydrophilicity [20]. Three-dimensional double-layer evaporation surfaces were formed by modulation of the yarn structure and fabric organization. During the evaporation process, the lower evaporation surface can recover the waste heat from the upper evaporation surface for water evaporation. The DLE’s surface increases the multi-level absorption and utilization of solar radiation. The three-dimensional flank structure can absorb heat from the environment for cold evaporation. The double-layer hollow structure can optimize the evaporation rate by utilizing wind energy. Through multi-level thermal management, the DLE can achieve efficient seawater desalination. Therefore, an outstanding evaporation rate of DLE 2.86 kg·m^−2^·h^−1^ is achieved under 1 sun intensity (1 kW·m^−2^). This DLE with excellent salt resistance demonstrates a competitive high-salinity (10 wt%) desalination rate (2.3 kg·m^−2^·h^−1^). After 5 days of outdoor testing, the average daily condensation collection can be maintained at around 8.9 kg·m^−2^, with a metal ion removal rate of up to 99%. Accordingly, the research and development of DLE may provide a new viable solution for the thermal management regulation of future solar desalination plants.

## 2. Experimental Section

### 2.1. Experimental Materials

Graphene oxide powders (GO, >95%,0.2–10 μm); DFM (AR); thermoplastic polyurethane (TPU, 58237); Tencel coarse yarns (100%, 750 tex); anhydrous ethanol (AR); methyl orange (AR); rhodamine B (AR); methyl Blue (AR); potassium chloride (AR).

### 2.2. Observation of Sample Morphology

A scanning field emission electron microscope (Regulus 8100, HITACHI, Tokyo, Japan) was used to observe the morphology and microstructure of double-layer photothermal fabrics (DLE). The fabrics were then dried and sprayed with gold. The tests were observed at 5.0 kV accelerating voltage after drying and spraying with gold for 90 s.

### 2.3. Wetting Performance Test

A contact angle measurement (OCS 40, Dataphysics, Stuttgart, Germany) was used to detect hydrophilicity. The samples were placed flat on a slide and 5 μL of pure water was dropped onto the surface of the specimen through a syringe; each sample was tested three times and averaged.

### 2.4. Characterization of Surface Functional Groups

The surface functional groups of the samples were characterized using Fourier transform infrared spectroscopy (Nicolet iS50, Thermo Scientific, Waltham, MA, USA) with a scanning range of 4000–500 cm^−1^ and a resolution of 2 cm^−1^.

### 2.5. Evaporation Performance Test

The photothermal fabric was first placed in a 250 mL beaker filled with an appropriate amount of water and placed in a polyethylene foam, which isolated the effect of the outside temperature on the evaporation properties. A xenon lamp (CELHXF300) (Au-light, Beijing, China) was used for indoor evaporation tests. The temperature of the evaporator at each stage of the evaporation process was determined by the infrared camera (FOTRIC 345) (FOTRIC, Shanghai, China) and multi-channel temperature recorder (SHSIWI TS-08A) (SHSIWI, Shanghai, China).

The evaporation rate Q can be calculated by Equation (1) [21,22]:

(1) $$Q=\frac{∆m}{S∆t}$$

In the above formula, Δm represents the evaporated mass of water during desalination (kg), S represents the evaporated area of the photothermal material (m^2^), and Δt represents the evaporation time (h).

### 2.6. Desalination and Outdoor Performance Test

Simulated seawater (NaCl concentrations of 3.5, 5, 10 wt%) was used to verify the desalination ability of DLE. This includes DLE’s salt crystallization self-cleaning test and mass change at 1 solar radiation at 8 h without applied airflow. To explore the usefulness of the evaporation equipment, an outdoor test setup was built. An ultra-transport acrylic Plexiglas cover was used for the condenser and Plexiglas vessels for water collection. The water for outdoor testing was taken from Dushu Lake in Suzhou and the Yellow Sea in China. The ion concentration of the desalinated pure water was measured by an s ICP spectrometer (ICPAP 6000) (Thermo Scientific, Waltham, MA, USA).

### 2.7. Preparation of DLE

#### 2.7.1. Preparation of GO Slurry

The 1 wt% GO dispersion was firstly prepared by adding GO powders into DMF by magnetic stirring for 4 h at 500 rpm. Subsequently, the obtained GO dispersion was impregnated in thermoplastic polyurethane (10 wt%) for bonding, and a homogeneous GO slurry was obtained by ultrasonication for 4 h.

#### 2.7.2. Preparation of GO Composite Yarn

Tencel roving was placed in a 1 wt% GO dispersion and sonicated for 20 min, then dried in an oven at 40 °C. The photothermal yarns were prepared by drafting and twisting the roving using the ring-spinning technique. Next, the GO slurry was passed at a speed of 20 m/min and placed in an oven at 60 °C for drying to obtain GO composite photothermal spun yarns. According to the team’s previous studies, the solar absorption of the GO-loaded Tencel yarn was significantly increased. The composite yarns are characterized by excellent heat conversion properties, moisture transport properties, and breathability and were obtained by combining and twisting GO-loaded yarns and Tencel yarns in a ratio of 3:1 [23]. In this research, the composite yarn was used to carry out practical applications.

#### 2.7.3. Preparation of Double-Layer Photothermal Fabric

**Figure 1:**
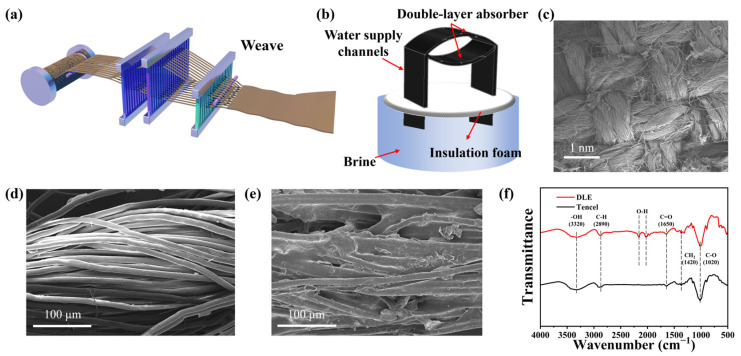
(**a**) Diagram of DLE weaving process, (**b**) schematic diagram of DLE, (**c**–**e**) SEM images of the DLE, (**f**) FTIR spectra of DLE and Tencel.

The GO composite yarns were prepared by weaving techniques to obtain two-dimensional photothermal fabrics (GOT-F). GO composite yarns were used as warp and weft yarns to manufacture DLE on a semi-automatic small sample loom using the smooth threading method. As shown in Figure 1b, an 8 cm satin-patterned self-supporting water transfer fabric was first woven. Secondly, a 6 cm double-layer plain weave photothermal conversion fabric organization was woven. Finally, a satin weave was woven to form a symmetrical DLE. The flat evaporator is called GOT-F. DLE was combined with polyethylene foam to form 3D evaporators, which were named DLE-H1, DLE-H2, DLE-H3, DLE-H4, and DLE-H5 for evaporator heights of 1, 2, 3, 4, and 5 cm, respectively. The evaporator opening heights were designed based on DLE-H4 and named DLE-D0 (DLE-H4), DLE-D1, DLE-D2, and DLE-D3 when the opening heights were 0, 1, 2, and 3 cm, respectively.

## 3. Results and Discussion

### 3.1. Characterization of Samples

The preparation process is shown in Figure 1a; three-dimensional double-layer photothermal fabrics (DLE) are prepared on a large scale by ring spinning as well as by the weaving method. In this case, GO is used as a photothermal conversion material, and Tencel is a hydrophilic material. The tubular fabric and polyethylene foam are assembled to form a 3D-integrated double-layer hollow structure evaporator (Figure 1b). The top is a plain double-layer absorber. The warp and weft yarns are interlaced with other threads with enhanced times of yarn bending and interlacing. The plain fabric has weak luster and lower light reflectivity. Both sides are satin water supply channels. Satin fabrics have twice as many warp yarns as double plain fabrics and are thick and stiff, forming the two wings of an arch structure for support and water supply. As can be seen from the SEM image, the Tencel fibers have a twisted helical structure (Figure 1c). Capillary channels are included between fibers and interlaced pores between yarns for water transport and steam passage [24]. From the SEM images at different magnifications, it is observed that the multi-stranded single yarns are twisted and held together with each other. The presence of irregular-sized TPU@GO particles on the surface of the yarn indicates successful loading. The SEM image of the pristine Tencel is shown in Figure 1d,e, which shows that the fiber surface is smooth. After GO and TPU loading, a large number of complexes are distributed on the surface of the rough Tencel. Meanwhile, the fiber structure remains intact. The rough Tencel surface reduces solar reflection and increases solar absorption [25]. Roughness improves the mechanical properties of fibers because of the increased adhesion between fibers [26].

The FTIR was performed to analyze the loading and interactions of functional groups on the DLE surface, as can be seen in Figure 1f. Compared to the pristine Tencel the DLE shows a new peak around 2000–2300 cm^−1^, which is due to the O-H stretching vibration formed by the GO particles [27]. Graphene oxide typically shows a broad hydroxyl peak at 3300–3500. This indicates a large number of hydroxyl groups on the DLE. The peaks near 3320, 2890, 1650, 1420, and 1020 cm^−1^ are attributed to -OH stretching vibrations, C-H asymmetric stretching, C=C stretching vibrations, CH2 symmetric bending, and C-O stretching, respectively [28,29]. The increased hydrophilic functional groups result in the strong water affinity of DLE. These hydrophilic functional groups are essential for water supply and diffusion. In addition, the pore structure of the fabric provides more water transport channels [30]. The successful loading of GO particles onto Tencel yarn is demonstrated.

**Figure 2:**
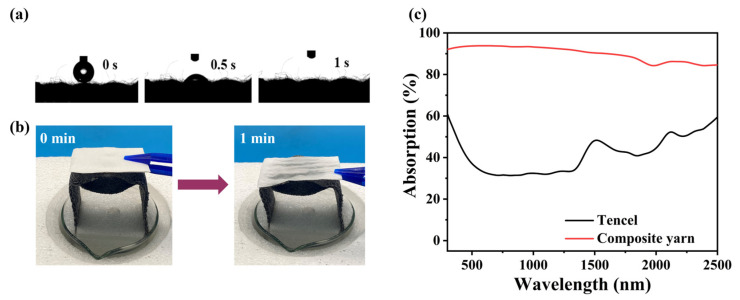
(**a**) Schematic of the water contact angle of the photothermal fabric DLE, (**b**) optical images of DLE water supply capacity, (**c**) solar absorption spectrogram.

Tencel fiber has good hydrophilic properties and capillary effect [31]. It acts as a substrate and water transport channel for DLE, ensuring an adequate supply of water and rapid transmission during evaporation. The yarn formed by adding GO to Tencel has excellent hydrophilicity, and water droplets can be absorbed within 1 s (Figure 2a). This distinguished hydrophilicity is due to the existence of GO-containing hydrophilic functional groups such as hydroxyl and carboxyl groups, and macro and micropores are formed between the fabrics [32]. The top and bottom sides of the DLE bilayer structure have the same parameters (GO loading, degree of twisting, hydrophilicity, etc.) except for the different fabric organization structures. The differently shaped pores create a capillary pressure difference, which facilitates moisture transport and absorption [33]. The water supply performance of the DLE is shown in Figure 2b, where a piece of dry white paper is placed on the top of the DLE, the white paper is wetted after 1 min, and the wet state could be maintained for 4 h. This fully demonstrates that DLE is capable of delivering moisture to the evaporating surface in a fast and continuous manner.

The absorption spectra in the UV-visible near-infrared range are shown in Figure 2c. Compared to Tencel, GO composite yarns exhibit excellent solar absorption in the full solar spectrum from 300–2500 nm, with a solar absorption rate of approximately 90%. This is because of GO’s inherent high solar absorption in a wide spectral range of photothermal conversion materials [34]. Thus, it can significantly increase the heat collection capacity of the DLE. The rough surface structure of the yarns and the pores between the fibers increase the reflected solar path between the yarns, further increasing the solar absorption [35].

### 3.2. Thermal Management and Evaporation

**Figure 3:**
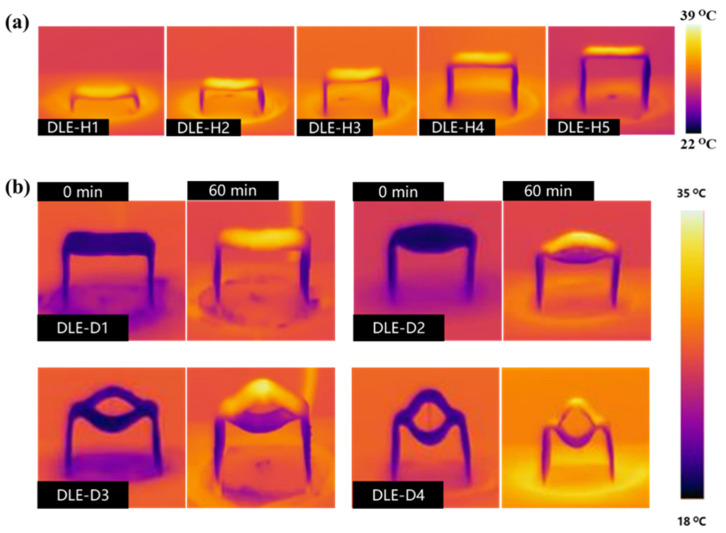
(**a**) Infrared thermal imaging of DLE-Hs after 60 min of operation under 1 sun solar radiation, (**b**) initial and steady-state infrared thermal imaging of DLE-Ds.

As shown in Figure 3a, abundant heat is generated on the top surface of DLE-Hs after 1 sun radiation. The flanks as water transport channels contain large amounts of water, which has a much greater specific heat capacity than air. The temperature of the flanks will therefore be lower than that of the environment, and thermal convection will be caused by the temperature difference [36]. Consequently, the DLE side absorbs additional heat from the environment for cold evaporation.

The surface temperature of DLE-Ds and the energy radiative transfer process between the upper and lower evaporating surfaces is shown in Figure 3b. The temperature difference in the double-opening structure reduces the exchange and loss of heat to the environment during heat transfer. Additionally, heat loss from the lower evaporating surface can also be absorbed by the upper for water evaporation.

**Figure 4:**
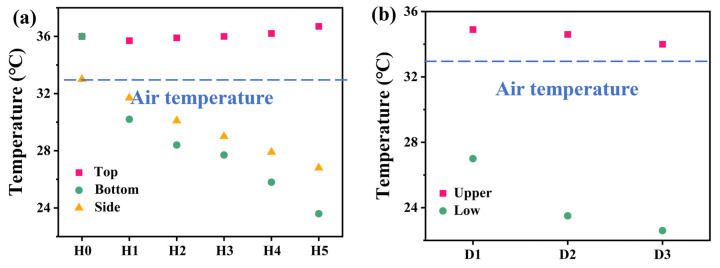
(**a**) The stable top, bottom, and side surface temperature of DLE-Hs under 1 sun radiation, (**b**) the stable upper and lower surface temperature of DLE-Ds under 1 sun radiation.

As shown in Figure 4, the steady-state temperatures of the top, sides, and bottom water of the DLE-Hs device are recorded using thermocouples after 60 min of operation under 1 sun radiation. As shown in Figure 4a, the side surface temperature increases with the height of DLE-Hs. The temperature difference between the side and the top becomes larger and larger, creating a large temperature difference. Simultaneously, the high temperature at the top conducts to the low temperature at the bottom, which can avoid heat loss. When the height is greater than 1 cm, the temperature of the flanks and bottom water is lower than the ambient temperature. The equipment absorbs additional energy from the environment, thus compensating for heat loss. As a result, the evaporation rate of a three-dimensional evaporator can be increased by 81% compared to a two-dimensional evaporator [23]. The temperature difference between the upper and lower surface of DLE-Ds for 60 min of operation was recorded by thermocouples. The differences of DLE-D1, DLE-D2, and DLE-D3 are 7.9, 11.1, and 11.4 °C (Figure 4b). The temperature of the upper surface is more stable, and the lower surface decreases with increasing height of the opening. The upper layer of the evaporator converts solar energy into heat and most of the heat is subsequently consumed as the enthalpy of the phase change of water [37]. The remaining heat is transferred to the flanks, while another part is transferred to the lower level for water evaporation by convection radiation, which improves the overall evaporation efficiency.

In terms of solar absorption, incident solar that has not been completely absorbed by the upper surface can be repeatedly absorbed and utilized by the lower surface. From the perspective of evaporation enhancement, in addition to the increase in evaporation area, the three-dimensional evaporator provides more space for vapors to escape, improving the overall evaporation efficiency. From the perspective of energy transfer, the energy of the upper and lower surfaces can complement each other and capture additional energy from the environment for multi-stage evaporation. Therefore, the height and openings of the DLE device may contribute to the overall evaporation rate.

**Figure 5:**
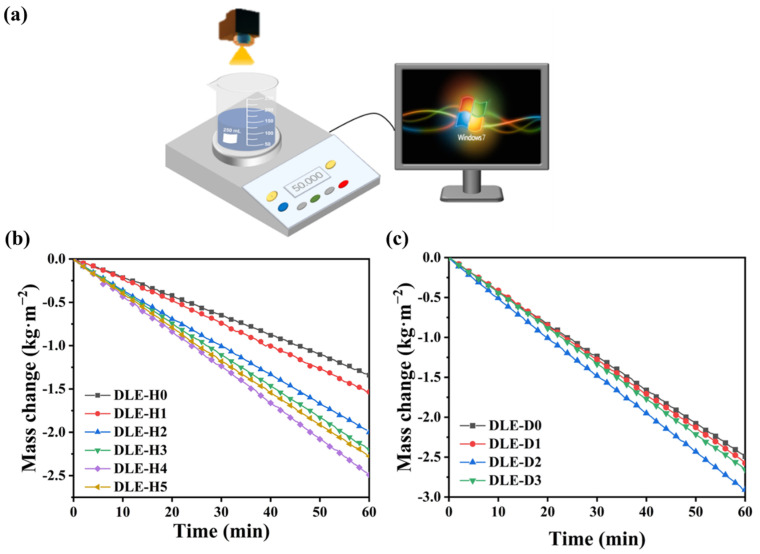
(**a**) Diagram of desalination performance testing device, mass change of (**b**) DLE-Hs after 60 min of operation and (**c**) DLE-Ds under 1sun radiation.

To investigate the effect of DLE device height and opening size on the evaporation rate, the evaporation rate of DLE with different device heights and opening sizes under 1 sun is tested here, as shown in Figure 5a. Firstly, the evaporation rate of DLE at different device heights is explored. The evaporation rate of DLE-Hs is shown in Figure 5b, the evaporation rates of DLE-H0 (GOT-F), DLE-H1, DLE-H2, DLE-H3, DLE-H4, and DLE-H5 are 1.37, 1.57, 1.96, 2.18, 2.48, and 2.23 kg·m^−2^·h^−1^. Compared with Tencel fiber, the evaporation rate of DLE has significantly increased [38]. GO is a carbon-based material, and due to the weak bonding strength of the π-bonds, electrons can be excited from π orbitals to π* orbitals at smaller energies, converting solar energy to heat through lattice vibrations [34]. GO loading increases the number of hydrophilic groups on the surface, ensures sufficient water transport, and accelerates evaporation [39]. In addition, the co-loading of TPU and GO increases the surface roughness and can increase light refraction. Compared with the planar two-dimensional structure, the maximum evaporation rate of DLE has increased by 81%. Due to the increase in the evaporation area of the three-dimensional evaporation system, the temperature of the flanks is lower than the ambient temperature, and the heat loss is reduced. It can capture heat from the environment to achieve cold evaporation, improving energy utilization efficiency and evaporation performance [40]. For DLE-H5, the water supply to the DLE is insufficient due to the increased distance of the water passages caused by the excessive height. The continuous water pathway between the bulk water and the porous medium is interrupted. When the heat is transferred downwards, the evaporation surface moves down to the interior of the medium and causes the rate of evaporation to decrease. Secondly, DLE-H4 is chosen to explore the effect of DLE opening size on evaporation rate. As shown in Figure 5c, the evaporation rates of DLE-H0, DLE-D1, DLE-D2, and DLE-D3 are 2.48, 2.58, 2.86, and 2.66 kg·m^−2^·h^−1^, respectively. Compared with DLE-H4 (DLE-H0), the evaporation rate of DLE-2 increases by 17%. On the one hand, because the arch structure of DLE increases the photothermal area and evaporative surface area, the actual solar area is larger than the projected area. On the other hand, the high temperature at the top is transferred to the bottom along the temperature gradient, which reduces heat loss in the environment. The residual heat of the upper evaporating surface can be absorbed by the lower evaporating surface through heat radiation, improving the efficiency of heat utilization. The heat convection of the evaporation process on the lower surface can be transferred upwards to the upper evaporation surface, which can further reduce the heat loss and improve the overall evaporation efficiency. The lower evaporation surface does not receive direct sunlight, although the upper surface consumes a lot of heat during evaporation. However, a lot of heat is transferred to the environment through thermal radiation, and the bottom surface has the same photothermal properties as the upper evaporating surface. The waste heat of the upper evaporating surface can be rationally utilized [41,42,43]. In addition, the surface of the upper evaporating surface contains pores, and some sunlight will pass through to the lower evaporating surface.

### 3.3. Wind Energy and Evaporation

**Figure 6:**
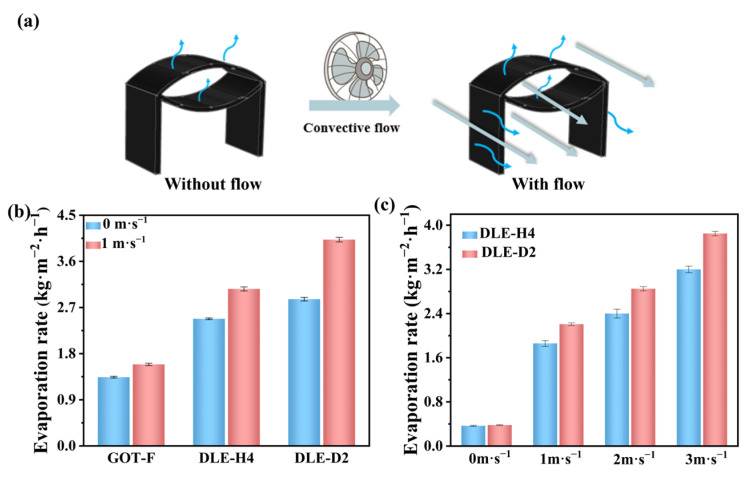
(**a**) Schematic of evaporation after loading wind energy, (**b**) evaporation rates of GOT-F, DLE-H4, DLE-D2 under 1 sun at low wind speeds, (**c**) evaporation rates of DLE-H4 and DLE-D2 under 1 sun at different wind speeds.

The evaporation performance of DLE is related to the environment, with ambient wind speed affecting evaporation performance by influencing moisture transport. The unpredictable environmental wind energy needs to optimize the design of the structure to make rational use of the wind energy. The effect of wind speed on the evaporative performance of the DLE is discussed in this part (Figure 6a).

GOT-F uses plain weave fabric as a planar structure, while DLE-H4 and DLE-D2 are compared as three-dimensional structures. As shown in Figure 6b, at a wind speed of 1 m·s^−1^, the enhancement is 1.19 times (1.59 kg·m^−2^·h^−1^), 1.24 times (3.07 kg·m^−2^·h^−1^), and 1.4 times (4.01 kg·m^−2^·h^−1^), respectively, compared to the wind-free condition. Compared to two-dimensional structures, three-dimensional structures not only have larger actual evaporation surfaces, but hollow structures can promote inner evaporation. DLE-D2 has a higher evaporation rate ratio than DLE-H4 in windy conditions. Because the DLE has a double-layer opening structure, it can further enhance the wind convection evaporation rate. The difference between the evaporation rates of DLE-D2 and DLE-H4 is expanded with the increase in wind speed. For the reason of inquiring into the effect of wind speed on the evaporation rate of DLE, the evaporation rates of DLE-D2 and DLE-H4 under dark conditions are tested at different wind speeds. As shown in Figure 6c, at the wind speeds of 0, 1, 2, and 3 m·s^−1^, the difference in evaporation rate between DLE-H4 and DLE-D2 is 1.04, 1.18, 1.19, and 2 times, respectively. The results indicate that the double-layer structure of DLE can enhance the improvement effect of wind on evaporation performance. When the airflow from a wide area flows into a narrow area, the density of the airflow increases, so it accelerates through the narrow area, and the wind speed in that area increases, known as the “canyon effect” [44]. As a result, the wind speed acting on the flanks and openings of the DLE-D2 becomes larger and the evaporation rate is enhanced.

### 3.4. The Stability Performance of DLE

**Figure 7:**
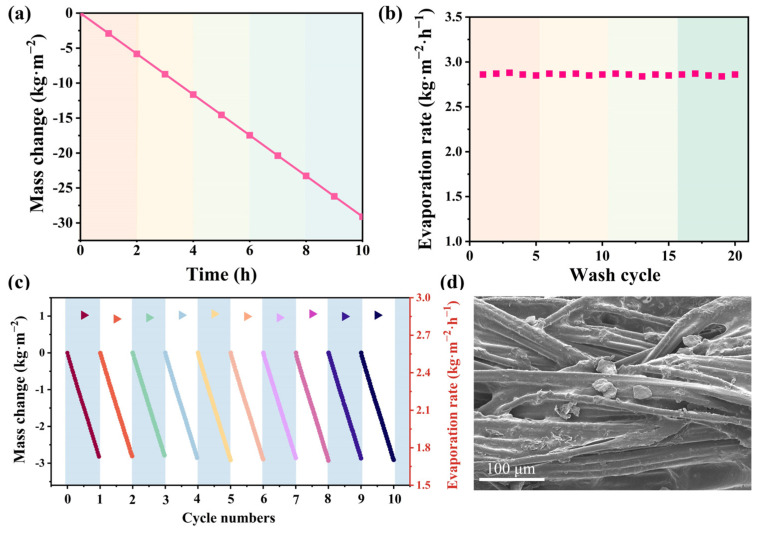
(**a**) Mass change of DLE-D2 run for 8 h under 1 sun radiation, (**b**) evaporation rate of DLE-D2 before and after 20 washes, (**c**) evaporation rate and mass change of DLE-D2 after 10 cycles under 1 sun radiation, (**d**) SEM image after friction treatment and long cycle.

In practice, in addition to excellent evaporation performance, evaporation stability is also critical for DLE. The water evaporation rate was always maintained at about 2.86 kg·m^−2^·h^−1^ (Figure 7a), indicating that DLE has a stable water supply capacity and evaporation performance. Next, the evaporation mass profile of DLE-2 remained almost unchanged after 20 ultrasonic washings, with the evaporation rate remaining above 96% of the initial value (Figure 7b). It suggests that because GO composite yarns are tightly bonded, photothermal loads are not easy to shed under complex conditions. GO particles can increase the gripping force between fibers and the bonding force between fibers and filaments. According to our previous research [23], fiber loaded with TPU has high tensile strength and strong encapsulation properties, allowing GO to be firmly adhered to the fibers. This outstanding stability is attributed to the adhesive effect of TPU as well as yarn homogenization and homogenization during the spinning process [25], which ensures the long-term operating capability of the evaporator. Lastly, the cycling performance of DLE-D2 is tested. After 10 consecutive cyclic evaporation tests, the DLE still maintains a stable and efficient water evaporation performance with an average rate of 2.85 kg·m^−2^·h^−1^ (Figure 7c). Fibrillation of the surface of Tencel is prone to occur. Increased fiber density during reprocessing of Tencel reduces fiber inhomogeneity [45]. Fibrillation increases the interfacial forces between TPU and GO, thus enhancing the bonding properties. It proves that DLE has excellent water evaporation stability and is highly practical. Tencel fibers are subjected to high friction after ring spinning. However, TPU with good adhesive properties is added to the fibers. The particles can then be firmly attached to the fibers and form an encapsulated structure. The particles remain intact after a long period of operation (Figure 7d).

### 3.5. Desalination Capacity of the DLE System

**Figure 8:**
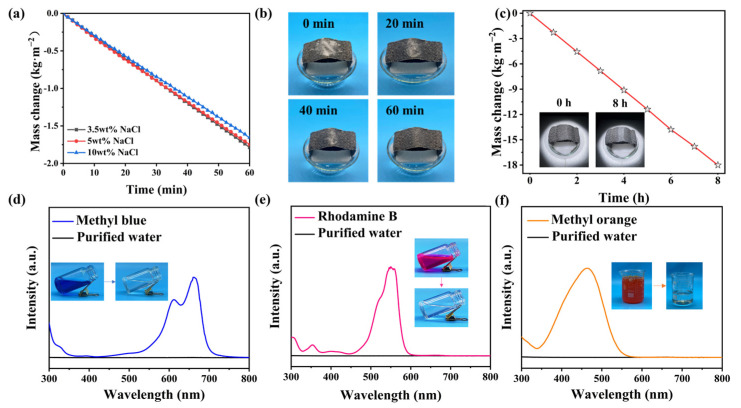
(**a**) Mass change of DLE-D2 in three different concentrations of brine under 1 sun radiation, (**b**) diagram of DLE self-cleaning, (**c**) mass change curve of DLE-2 running in saline water for 8 h under a solar intensity of 1 kw·m^−2^, (**d**–**f**) ultraviolet-visible absorption spectra of dye wastewater treated with DLE.

The DLE needs to maintain good desalination capability in high-salt-concentration brine. Accordingly, DLE-D2 was selected to test the desalination performance in different salt concentrations. The evaporation rates of DLE-D2 in NaCl concentrations of 3.5, 5, and 10 wt% are 2.57, 2.38, and 2.28 kg·m^−2^·h^−1^, respectively (Figure 8a). The evaporation rates are 89, 83, and 90% of the pure water. The competitive desalination rates in brines of varied concentrations demonstrate the outstanding salt resistance of the DLE. This is due to the high porosity between the fabrics and the capillary action between the yarns which contribute to the rapid transport of moisture and the diffusion of salt particles [46]. In addition, the DLE evaporator is characterized by its salt removal and self-cleaning capabilities. The experiments were carried out by placing 1.0 g of NaCl crystals on the upper evaporation surface of the DLE and recording the dissolution of the salt crystals over different periods. After 60 min of illumination, the salt crystals on the evaporation surface were almost completely dissolved (Figure 8b). This indicated that the DLE has outstanding salt removal and self-cleaning capabilities. The mass change curve of the DLE running in low-concentration saline water for 8 h is shown in Figure 8c, and the evaporation rate remains stable. The illustration shows no visible crystal precipitation. Finally, the performance of DLE wastewater treatment is explored as shown in Figure 8d–f. After the treatment of dye waste liquid rhodamine B, methyl blue, and methyl orange with DLE-D2, the resulting clean water is colorless and transparent. The unique characteristic peaks of dyes disappeared, which proved that DLE has a certain capacity for effluent purification.

### 3.6. Outdoor Desalination Test

**Figure 9:**
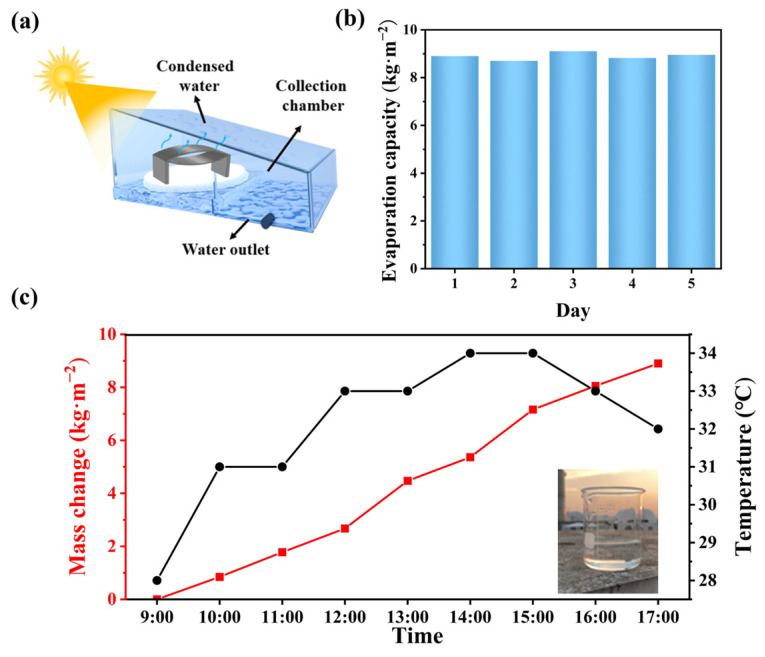
(**a**) Schematic diagram of outdoor test with DLE-D2, (**b**) evaporation capacity during outdoor testing for 5 days, (**c**) cumulative mass change during a continuous test from 9:00 to 17:00.

A 10 cm × 10 cm DLE integrated evaporator placed in an ultra-transparent acrylic Plexiglas condenser with a removable lid was prepared for outdoor desalination testing as shown in Figure 9a. Figure 9b shows the testing of the evaporation durability through a 5-day seawater desalination experiment. The evaporation and condensation performance of the DLE remains competitive, with evaporation rates fluctuating up and down due to weather variations, but average evaporation remains around 8.9 kg·m^−2^. Long-term stable evaporation properties are obtained because the roving is constantly subjected to stretching, elongation, and twisting during the ring-spinning process. It promotes uniform loading of GO particles inside the yarn [47]. The hydrophilic characteristic of GO composite yarns allows brine to advent and diffuse through the double-layered fabric, which alleviates the deposition of salt particles and impurity ions. This increases durability and ion removal efficiency [48]. Seawater from the Yellow Sea in China was used for the outdoor test, and the outdoor experiment was conducted from 9:00 to 17:00. The cumulative evaporation from the DLE was 8.9 kg·m^−2^ (Figure 9c), and approximately about 100 mL of clean water could be collected as shown in the inset. The TPU-covered fabric surface contains a large roughness that reduces the refraction of light and increases the absorption of sunlight. The special structure of the double-opening fabric allows for the absorption of heat from the environment as well as the multiple uses of heat [49]. To explore the seawater purification ability of DLE, the concentrations of major ions (Ca^2+^, Mg^2+^, Na^+^, and K^+^) in seawater before and after desalination were measured. After purification, the ion concentrations in the purified water were 5.096, 9.726, 76.89, and 6.377 mg·L^−1^, respectively, and the ion removal rate reached about 99.5%. GO functional groups can bind to hydrated metal ions via electrostatic attraction, ion exchange, and metal-π interactions [23,50]. The concentration of ions in clean water meets World Health Organization drinking water standards [51]. In a word, DLE evaporators have excellent desalination and purification capabilities in outdoor environments.

## 4. Conclusions

In this research, photothermal composite yarns were prepared by impregnation, fusion, and twisting textile processes using GO as the photothermal material and Tencel as the substrate material. This impregnation method ensures that GO particles can be uniformly loaded into the interior and surface of the yarn. GO particles can be tightly wrapped with Tencel due to the existence of TPU coverage. Adjustable twisting technology is used to regulate the ratio of GO to Tencel fibers to achieve a dynamic balance between water supply and heat. Overall, the preparation of three-dimensional photothermal fabrics by fabric organization design and weaving process exhibits unique practicality, employing three-dimensional double-layer evaporation surface fabrics with a hollow arch by the design of the regulating structure. This increases the actual solar area, enlarges the multi-stage use of solar energy, and improves the thermal energy utilization of the evaporator. During evaporation, waste heat from the upper surface can be transferred to the lower surface by convective radiation. Due to the temperature difference, the flanks can capture additional heat from the environment to achieve cold evaporation, reducing heat loss from the top to the bottom. The double-layered hollow structure enhances wind energy utilization for evaporation acceleration. As a result, DLE evaporation rates of up to 2.86 kg·m^−2^·h^−1^ under 1 sun radiation were recorded. The evaporation rate is maintained at 2.25 kg·m^−2^·h^−1^ after 8 h of operation in 10 wt% high-concentration brine. This stable and outstanding performance proves the practical application abilities of DLE.

## Data Availability

The data presented in this study are available on request from the corresponding author due to privacy.
